# The epidemiology of patellar luxation in dogs attending primary-care veterinary practices in England

**DOI:** 10.1186/s40575-016-0034-0

**Published:** 2016-06-08

**Authors:** Dan G. O’Neill, Richard L. Meeson, Adam Sheridan, David B. Church, Dave C. Brodbelt

**Affiliations:** Production and Population Health, The Royal Veterinary College, Hawkshead Lane, North Mymms, Hatfield, Herts AL9 7TA UK; Clinical Sciences and Services, The Royal Veterinary College, Hawkshead Lane, North Mymms, Hatfield, Herts AL9 7TA UK

**Keywords:** Patellar luxation, VetCompass, Electronic patient records, Breed, Dog, Epidemiology, Primary care, Veterinary

## Abstract

**Background:**

Canine patellar luxation is one of the most common orthopaedic disorders of dogs and is a potential welfare concern because it can lead to lameness, osteoarthritis and pain. However, there are limited epidemiological data on the disorder relating to the general population of dogs in England. This study aimed to investigate the VetCompass Programme database of dogs attending primary-care veterinary practices in England to report on the prevalence, risk factors and clinical management of diagnosed patellar luxation cases.

**Results:**

The study included all dogs with at least one electronic patient record in the VetCompass database from September 1^st^, 2009 to August 31^st^, 2014. Candidate patellar luxation cases were identified using free-text word searching of the clinical notes and VeNom diagnosis term fields. Univariable and multivariable binary logistic regression modelling was used for risk factor analysis.

The overall dataset comprised 210,824 dogs attending 119 clinics in England. The prevalence of patellar luxation diagnosis in dogs was 1.30 % (95 % confidence interval (CI) 1.21–1.39). Of the 751 incident cases, 293 (39.0 %) received medical management, 99 (13.2 %) received surgical intervention and 28 (3.7 %) were referred for further management.

Multivariable modelling documented 11 breeds with increased odds of patellar luxation compared with crossbred dogs, including the Pomeranian (odds ratio [OR]: 6.5, 95 % CI 4.0–10.7, *P* < 0.001), Chihuahua (OR: 5.9, 95 % CI 4.4–7.9, *P* < 0.001), Yorkshire Terrier (OR: 5.5, 95 % CI 4.3–7.1, *P* < 0.001) and French Bulldog (OR: 5.4, 95 % CI 3.1–9.3, *P* < 0.001). Dogs with bodyweight below their mean for breed and sex had a 1.4 times odds of diagnosis (95 % CI 1.2–1.6, *P* < 0.001). Dogs aged ≥ 12.0 years showed 0.4 times the odds (95 % CI 0.3–0.5, *P* < 0.001) compared with dogs aged < 3.0 years. Females had 1.3 times the odds (95 % CI 1.1–1.5, *P* < 0.001), neutered dogs had 2.4 times the odds (95 % CI 1.8–3.2, *P* < 0.001) and insured dogs had 1.9 times the odds (95 % CI 1.6–2.3, *P* < 0.001).

**Conclusions:**

Patellar luxation warrants inclusion as a welfare priority in dogs and control strategies that include this disorder should be considered as worthwhile breeding goals, especially in predisposed breeds.

## Plain english summary

Patellar luxation (slipping kneecap) describes a condition where the kneecap (patella) can dislocate (slip) from its normal groove at the bottom of the thighbone (femur). This may be painless initially but can progress to arthritis later, causing pain and chronic lameness. Affected dogs are typically born normal but develop leg problems as they grow, which can allow the kneecap to slip abnormally in and out of its groove in the knee joint. This prevents the dog from bending its knee and can cause severe friction and rubbing of the surfaces of the joint, leading to arthritis. For some dogs, the kneecap remains permanently out of the groove giving permanent disability. Although patellar luxation is believed to be common in dogs in England and to be more common in certain dog breeds, particularly small breeds, precise information for the general population of dogs in England is lacking. The VetCompass Programme collects de-identified medical records from veterinary clinics in the UK to allow investigation of health conditions in dogs.

In this study of 210,824 dogs attending 119 veterinary clinics in England, patellar luxation affected 1.3 % of dogs overall. These dogs were managed in different ways; 39 % were treated with medication, 13 % had surgery and 4 % were referred for specialist veterinary management. The Pomeranian, Chihuahua and French Bulldog were particularly predisposed to patellar luxation. Dogs that were female, neutered (castrated or spayed) or below the average weight for their breed were at increased odds of diagnosis. Dogs that were covered by pet insurance were also more likely to be diagnosed. Most dogs were young when first diagnosed.

This is the largest study of its kind looking at patellar luxation in dogs attending veterinary general practices in England. It is clear that many dogs are affected and that there are breed-related genetic/inherited factors that predispose to patellar luxation. The welfare impact of patellar luxation on dogs in England should be taken into consideration in breeding programmes of commonly affected breeds.

## Background

Canine patellar luxation has been recognised as an important joint deformity in dogs for over half a century [1] and is reported to be one of the most common orthopaedic disorders currently seen in dogs [2–4]. Characterised by abnormal medio-lateral movement of the patella outside the confines of the trochlear sulcus [3, 4], patellar luxation is considered a developmental disease which is not present at birth but develops from a young age [4, 5], suggesting both environmental and heritable influences [5]. Patellar luxation is of welfare and clinical significance because it can lead to lameness, osteoarthritis and pain [6–8]. In consequence, a range of surgical techniques have been described to manage this condition [3, 7, 9–20].

Although patellar luxation has been reported to be one of the five most important hereditary defects in dogs from a welfare impact perspective [21, 22], and the seventh most common orthopaedic condition, seen in dogs [2], there are limited epidemiological data available on the prevalence, management and risk factors for the condition in the general population of dogs in England. The majority of published prevalence estimates for patellar luxation in dogs were based on US referral populations from university hospitals which have reported prevalence values ranging from 1.5–8.0 % [5, 21]. However, it is well recognised that referral populations can differ greatly to the wider general population and therefore may be an unreliable data source for generalisable prevalence estimates [23, 24]. Furthermore, management methods reported from referral studies reflect the preferred approaches of these specialist centres and will not include those cases treated conservatively in the primary-care setting. Some studies have attempted to report on risk factors for patellar luxation but many have lacked a control population and thus are difficult to interpret [2, 7, 10, 17, 21, 25, 26]. Historically, patellar luxation has been most associated with smaller-size dogs, with smaller breeds reported to have up to 12 times the risk compared with larger breeds [4, 21, 27, 28]. The Orthopaedic Foundation for Animals (OFA) surveys orthopaedic disease in animals voluntarily submitted for evaluation in the US [29]. It ranked the Pomeranian and Yorkshire Terrier as the two most commonly affected breeds with 37.2 % and 24.1 % of submissions affected respectively and has reported small breed dogs in general as having the highest prevalence of patellar luxation [30]. A study based on Veterinary Medical Database (VMDB) clinical data from US veterinary teaching hospitals identified the Pomeranian, Silky Terrier and Miniature Pinscher as breeds at substantially increased risk [5]. Recently, however, some studies have suggested that larger breeds, and in particular the Labrador Retriever, may now increasingly be affected [7, 17, 26]. Many of these studies, however, were based on relatively small cohorts (often 150 or fewer dogs), voluntary submissions or referral caseloads and, consequently, these study populations may be less representative of the true overall demographic than caseloads seen by primary-care practices. The former studies may reflect biased inclusion of certain subsets of patellar luxation caseloads such as larger body-size, insured, purebred or more severely affected dogs [23]. Furthermore, the literature is heavily biased towards reporting case series of patellar luxation cases that are mainly managed using surgical techniques [7, 9–20] where the absence of an explicit comparison group of unaffected animals prevents distinction between whether commonly affected breeds are truly at increased risk or are just more common in the dog population or more commonly referred [31]. Systematic collection and interrogation of a large merged database of primary-care clinical data would counter many of these limitations by enabling evaluation of patellar luxation within a large group of both affected and non-affected primary care dogs and would therefore be more representative of the general population of dogs [24].

This study aimed to investigate patellar luxation diagnosis in dogs attending primary-care veterinary practices in England by exploring the VetCompass Programme database [32]. The study objectives included estimation of the prevalence of patellar luxation diagnosis and evaluation of breed, sex, neuter status, bodyweight, age and insurance as risk factors for diagnosis. A further objective of the study was to report on current clinical management of all diagnosed patellar luxation cases. The study hypothesised that smaller breed dogs are predisposed to patellar luxation despite some recent reports that emphasise the disorder in larger breeds [7, 17, 26].

## Methods

The VetCompass Programme [32] collates de-identified electronic patient record (EPR) data from primary-care veterinary practices in the UK for epidemiological research [33]. Collaborating practices were selected by their willingness to participate and their recording of clinical data within an appropriately configured practice management system. Practitioners could record summary diagnosis terms from an embedded VeNom Code list [34] during episodes of care. Information collected included patient demographic (species, breed, date of birth, sex, neuter status, insurance status and bodyweight) and clinical information (free-form text clinical notes, summary diagnosis terms and treatment, with relevant dates) data fields. The background and general methodology for VetCompass data collection and studies have been described previously [33].

A cross-sectional study design using cohort clinical data was used to estimate the prevalence and risk factors for patellar luxation diagnosis. The sampling frame for the current study included all dogs with at least one EPR (clinical note, VeNom summary term, bodyweight or treatment) uploaded to the VetCompass database from September 1^st^, 2009 to August 31^st^, 2014. Sample size calculations estimated a cross-sectional study with 6,728 dogs weighing < 10 kg and 26,911 dogs weighing ≥ 10 kg would have 90 % power to identify small bodyweight as a risk factor with an odds ratio of ≥ 1.5 (α = 0.05) assuming a 1.0 % prevalence in the larger bodyweight group [35]. Ethical approval of the project was granted by the RVC Ethics and Welfare Committee (reference number 2015 1369).

The case definition for patellar luxation required a definitive diagnosis recorded in the EPR of patellar luxation (or synonym) that was based on physical and/or radiological examination in either a conscious or sedated animal. Animals with evidence that the patellar luxation resulted directly from a traumatic incident were excluded. Case-finding involved a two-step process of initial EPR screening followed by case verification by manually reading the EPRs. No cases were accepted based on free-text searching alone. Initial screening of all EPRs for candidate patellar luxation cases involved searching the clinical free-text field (search terms: *patella, MPL, LPL, PL, slipping pat, floating pat, trochlear groove, kneecap lux, femoral groove, floating kneecap, slipping kneecap, kneecap disloc*) and the VeNom term field (*patellar luxation, patella luxation*). The candidate cases identified from these searches were merged and the full clinical notes of a random subset were manually reviewed to decide on case inclusion. Randomisation used the *RAND* function in Microsoft Excel (Microsoft Office Excel 2007, Microsoft Corp.). Additional data were extracted on confirmed patellar luxation cases that described whether the case was pre-existing (first recorded prior to the study period) or incident (first recorded during the study period), the date of first diagnosis (for incident cases only), limb(s) affected (left, right, bilateral), whether and when surgery was performed, whether the animal was referred for advanced case management and whether any non-surgical approaches to management were used, such as analgesics, disease modifying agents, or neutraceuticals (not including commercial diets with chondroprotectant formulations) as part of case management with the aim of either preventing/delaying joint deterioration or reducing the inflammatory sequelae from the patellar luxation disorder. All dogs that were not identified as candidate patellar luxation cases during the initial screening were included as non-cases in the risk factor analysis.

A *breed* variable included individual breeds with 15 or more patellar luxation cases, a grouped category of all remaining breeds and a general grouping of crossbred dogs. A *purebred* variable categorised all dogs with a recognisable breed name as ‘purebred’ and the remaining dogs as ‘crossbred’ [36]. A *Kennel Club breed group* variable classified breeds recognised by the UK Kennel Club into their relevant breed groups (gundog, hound, pastoral, terrier, toy, utility and working) and all remaining dogs were classified as non-Kennel Club recognised [37]. *Neuter* described the status of the dog (neutered or entire) recorded at the final EPR. *Insurance* described whether a dog was insured at any point during the study period. The age value described the age at the date of first diagnosis for incident patellar luxation cases and was entered as unknown for pre-existing patellar luxation cases. For non-case dogs, the age described the age at the mid-point between the dates of the first and final EPRs recorded during the study period. This approach enabled the risk factor results to be interpreted as showing the odds at each age group for ‘becoming a case’ rather than for ‘being a case’. *Age* (years) was categorised into six groups (<3.0, 3.0–5.9, 6.0–8.9, 9.0–11.9, ≥ 12.0, not recorded). *Adult bodyweight* described the maximum bodyweight recorded during the study period for dogs older than 18 months and was categorised into six groups (0.0–9.9 kg, 10.0–19.9 kg, 20.0–29.9 kg, 30.0–39.9 kg, ≥ 40.0 kg, not recorded). The mean bodyweight of dogs older than eighteen months was calculated for each breed and sex, and used to generate a *breed-relative bodyweight* variable that characterised individual dogs as either below or equal/above the mean bodyweight for their breed and sex. This variable allowed the effect of adult body weight to be assessed *within* each breed and sex. The time contributed to the study for each dog described the period from the dates of the earliest to the latest EPR.

Following data checking and cleaning in Excel (Microsoft Office Excel 2013, Microsoft Corp.), analyses were conducted using Stata Version 13 (Stata Corporation). The period prevalence with 95 % confidence intervals (CI) described the probability of dogs having patellar luxation diagnosed at any time during the study period and included dogs that were diagnosed with patellar luxation prior to the study period (pre-existing cases) as well as those diagnosed for the first time during the study period (incident cases). Because the sampling design involved manual verification of a subset of the candidate cases, the overall prevalence of patellar luxation diagnosis was estimated using the Stata *survey* function and assigned probability weightings that denoted the inverse of the probability that each sampled observation was included [38]. The CI estimates were derived from standard errors, based on approximation to the normal distribution [39]. Descriptive statistics characterised the purebred status, breed, Kennel Club breed group, sex, neuter status, insurance, age, adult bodyweight, breed-relative bodyweight and time contributed to the study for the case and non-case dogs. Clinical management regimes were reported for incident cases only.

Binary logistic regression modelling was used to evaluate univariable associations between risk factors (*purebred, breed, Kennel Club breed group, adult bodyweight, breed-relative bodyweight, age, sex, neuter* and *insurance*) and a diagnosis of patellar luxation. Both pre-existing and incident patellar luxation cases were included in risk factor analysis. Because breed was a factor of primary interest for the study, *purebred* and *Kennel Club breed group* (both variables collinear with breed) and *adult bodyweight* (a defining characteristic of individual breeds) were excluded from multivariable modelling and instead univariable analysis results were interpreted for these variables. Remaining risk factors with liberal associations in univariable modelling (*P* < 0.2) were taken forward for multivariable evaluation. Model development used manual backwards stepwise elimination. Clinic attended was evaluated as a random effect and pair-wise interaction effects were evaluated for the final model variables [31]. The area under the ROC curve and the Hosmer-Lemeshow test were used to evaluate the quality of the model fit and discrimination (non-random effect model) [31, 40]. Statistical significance was set at *P* < 0.05.

## Results

The overall dataset comprised 210,824 dogs attending 119 clinics in England. Of the 6,340 candidate animals identified for patellar luxation, 1,998 (31.5 %) were manually checked to verify their case status and 854 dogs met the case definition for patellar luxation (42.7 % of candidate animals were confirmed as cases). After excluding the 4,342 candidate animals that were not manually checked, there were 206,482 animals included in risk factor analysis. The median (interquartile range [IQR], range) time contributed to the study for each dog was 0.6 years (0.0–2.1, 0.0–5.0).

After accounting for the effects of the subsampling protocol, the estimated prevalence for patellar luxation diagnosis in dogs overall was 1.30 % (95 % confidence interval (CI) 1.21–1.39). The breed prevalence results indicated that the breeds with the highest prevalence of patellar luxation diagnosis included the Pomeranian (6.5 %, 95 % CI 3.9-9.2), Yorkshire terrier (5.4 %, 95 % CI 4.4–6.3) and Chihuahua (4.9 %, 95 % CI 3.8–6.0). The prevalence in crossbreds was 1.2 % (95 % CI 1.0–1.4) (Table 1).

**Table 1:** Prevalence of patellar luxation diagnosed in commonly affected dog breeds attending primary-care veterinary practices in England

Breed	No. dogs in study	Prevalence %	95 % CI^a^
Pomeranian	1040	6.5	3.9–9.2
Yorkshire Terrier	6980	5.4	4.4–6.3
Chihuahua	4619	4.9	3.8–6.0
French Bulldog	1280	4.0	2.1–5.8
Lhasa Apso	1686	3.8	2.2–5.4
Cavalier King Charles Spaniel	4461	3.8	2.8–4.8
Bichon	2839	3.8	2.5–5.0
Pug	1998	3.5	2.1–4.9
Bulldog	1786	2.9	1.5–4.2
West Highland White Terrier	5275	2.5	1.7–3.2
Jack Russell Terrier	13520	1.7	1.3–2.1
Shih-tzu	4151	1.4	0.8–2.0
Crossbreed	47300	1.2	1.0–1.4
Staffordshire Bull Terrier	16746	0.5	0.3–0.7

^a^CI confidence interval

**Fig. 1:**
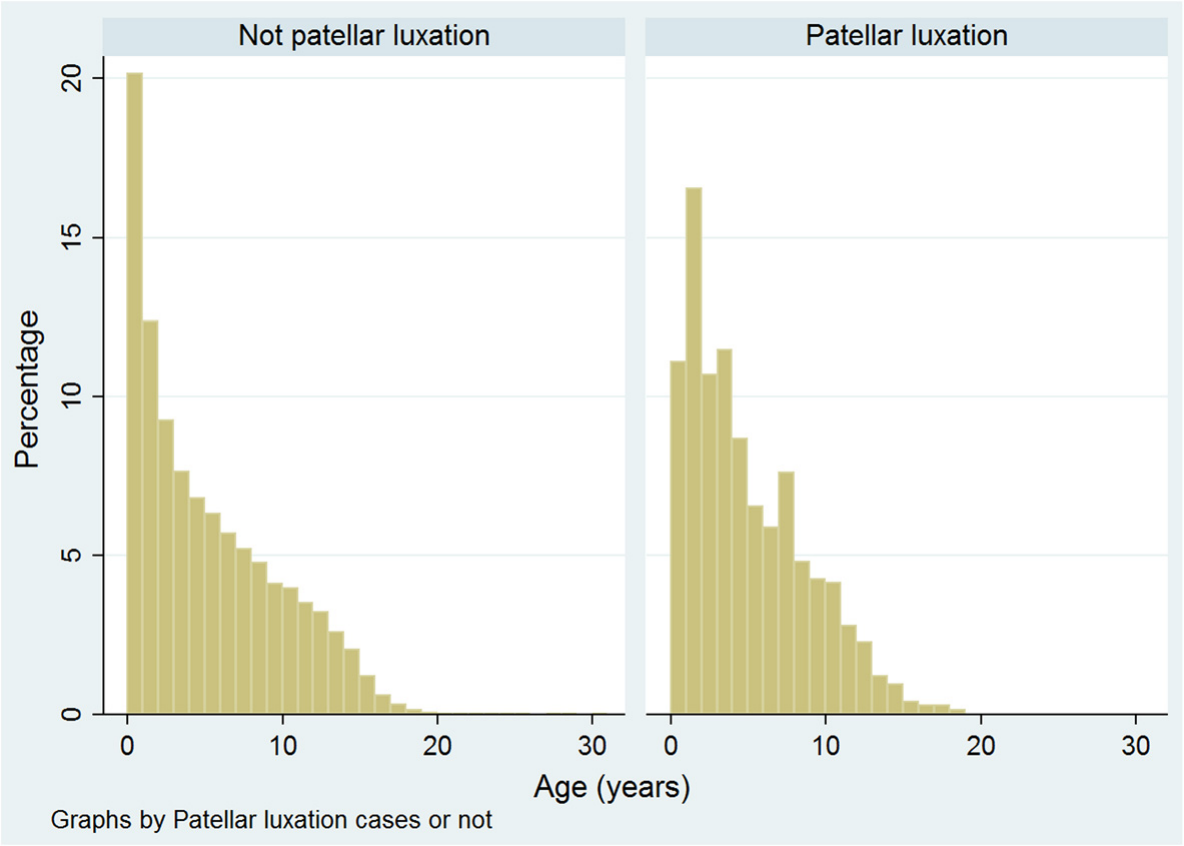
Ages of dogs attending primary-care veterinary practices in England without (*n* = 205,105) and with (*n* = 749) a patellar luxation diagnosis. The age was calculated for non-cases at the centre-date of the available clinical records and for cases at the date of first diagnosis

Data completion overall varied between the variables assessed: breed 99.9 %, age 99.7 %, sex 99.5 %, adult bodyweight (aged > 18 months) 59.4 %, insurance 58.0 %, and neuter 46.4 %. Of the patellar luxation cases with information available, 680 (79.6 %) were purebred, 468 (54.8 %) were female, 513 (88.0 %) were neutered and 375 (58.2 %) were insured. The median adult bodyweight of cases was 8.0 kg (IQR: 5.2–11.2, range: 1.4–65.5) and the median age at diagnosis was 4.0 years (IQR: 1.7–7.5 range: 0.2–19.0) years (Fig. 1). The most common breeds diagnosed with patellar luxation were the Yorkshire Terrier (*n* = 116, 13.6 % of all cases), Chihuahua (70, 8.2 %), Jack Russell Terrier (70, 8.2 %) and Cavalier King Charles Spaniel (52, 6.1 %), as well as 174 (20.4 %) crossbreds (Table 2).

**Table 2:** Descriptive and univariable logistic regression results for risk factors associated with patellar luxation diagnosis in dogs attending primary-care veterinary practices in England

Variable	Category	Case No. (%)	Non-case No. (%)	Odds ratio	95 % CI	*P*-value
Purebred status	Crossbred	174 (20.4)	46291 (22.5)	Base		
	Purebred	680 (79.6)	159205 (77.5)	1.1	1.0–1.3	0.133
Common breed-types	Crossbred	174 (20.4)	46291 (22.5)	Base		
	Yorkshire Terrier	116 (13.6)	6481 (3.2)	4.8	3.8–6.0	<0.001
	Chihuahua	70 (8.2)	4323 (2.1)	4.3	3.3–5.7	<0.001
	Jack Russell Terrier	70 (80.2)	13097 (6.4)	1.4	1.1–1.9	0.013
	Cavalier King Charles Spaniel	52 (6.1)	4183 (2.0)	3.3	2.4–4.5	<0.001
	West Highland White Terrier	40 (4.7)	5025 (2.5)	2.1	1.5–3.0	<0.001
	Bichon	33 (3.9)	2686 (1.3)	3.3	2.2–4.8	<0.001
	Staffordshire Bull Terrier	26 (3.0)	16458 (8.0)	0.4	0.3–0.6	<0.001
	Pug	22 (2.6)	1933 (0.9)	3.0	1.9–4.7	<0.001
	Pomeranian	21 (2.5)	951 (0.5)	5.9	3.7–9.3	<0.001
	Lhasa Apso	20 (2.3)	1605 (0.8)	3.3	2.1–5.3	<0.001
	Shih-tzu	18 (2.1)	4053 (2.0)	1.2	0.7–1.9	0.501
	Bulldog	16 (1.9)	1728 (0.8)	2.5	1.5–4.1	0.001
	French Bulldog	16 91.9)	1230 (0.6)	3.5	2.1–5.8	<0.001
	Other breed-types	160 (18.7)	95452 (46.5)	0.4	0.4–0.6	<0.001
Kennel Club Breed Groups	Not Kennel Club recognised	251 (29.4)	62010 (30.2)	Base		
	Toy	354 (41.5)	23937 (11.6)	3.7	3.1–4.3	<0.001
	Utility	107 (12.5)	16872 (8.2)	1.6	1.2–2.0	<0.001
	Terrier	89 (10.4)	28772 (14.0)	0.8	0.6–1.0	0.030
	Gundog	26 (3.0)	38001 (18.5)	0.2	0.1–0.3	<0.001
	Hound	13 (1.5)	9134 (4.4)	0.4	0.2–0.6	<0.001
	Pastoral	7 (0.8)	15403 (7.5)	0.1	0.1–0.2	<0.001
	Working	7 (0.8)	11367 (5.5)	0.2	0.1–0.3	<0.001
Adult (>18 months) bodyweight (kg)	<10.0	473 (55.4)	32,604 (15.9)	2.8	2.3–3.3	<0.001
	10.0–19.9	167 (19.6)	32,218 (15.7)	Base		
	20.0–20.9	39 (4.6)	27,233 (13.2)	0.3	0.2–0.4	<0.001
	30.0–30.9	11 (1.3)	19,384 (9.4)	0.1	0.1–0.2	<0.001
	≥40.0	11 (1.3)	9,409 (4.6)	0.2	0.1–0.4	<0.001
	Not recorded	153 (17.9)	84,780 (41.2)	0.4	0.3–0.4	<0.001
Breed relative bodyweight^a^	Equal/Higher	283 (33.1)	55,494 (27.0)	Base		
	Lower	418 (49.0)	65,355 (31.8)	1.3	1.1–1.5	0.003
	Not recorded	153 (17.9)	84,779 (41.1)	0.4	0.3–0.4	<0.001
Age category (years) (pre-existing cases included as non-recorded)	<3.0	287 (33.6)	85665 (41.7)	Base		
	3.0 – 5.9	200 (23.4)	42541 (20.7)	1.4	1.2–1.7	<0.001
	6.0 – 8.9	137 (16.0)	32179 (15.7)	1.3	1.0–1.6	0.021
	9.0 – 11.9	84 (9.8)	23819 (11.6)	1.1	0.8–1.3	0.680
	≥12.0	41 (4.8)	20901 (10.2)	0.6	0.4–0.8	0.001
	Not recorded	105 (12.3)	523 (0.3)	59.9	47.2–76.1	<0.001
Sex	Male	385 (45.1)	106869 (52.2)	Base		
	Female	468 (54.8)	97768 (47.8)	1.3	1.2–1.5	<0.001
Neuter status	Entire	70 (8.2)	20650 (10.0)	Base		
	Neutered	513 (60.1)	73594 (35.8)	2.1	1.6–2.6	<0.001
	Not recorded	271 (31.7)	111384 (54.2)	0.7	0.6–0.9	0.014
Insurance	Non-insured	269 (31.5)	70794 (34.4)	Base		
	Insured	375 (43.9)	47507 (23.1)	2.1	1.8–2.4	<0.001
	Not recorded	210 (24.6)	87327 (42.5)	0.6	0.5–0.8	<0.001

Data on both pre-existing and incident patellar luxation cases are included

Breed relative bodyweight^a^ for maximum bodyweight recorded in dogs aged over 18 months compared to the mean bodyweight of all study dogs for breed and sex

Of the non-case dogs with information available, 159,205 (77.5 %) were purebred, 97,768 (47.8 %) were female, 73,594 (78.1 %) were neutered and 47,507 (40.2 %) were insured. The median bodyweight was 18.4 (IQR: 5.9–29.3, range: 0.8–136.0) kg and the median age was 4.0 (IQR: 1.3–8.1, range: 0.0–30.8) years. The most common breeds among the non-case dogs were the Labrador Retriever (17,589, 8.6 %), Staffordshire Bull Terrier (16,353, 8.0 %), Jack Russell Terrier (13,097, 6.4 %) and Cocker Spaniel (7,581, 3.7 %) as well as 39,620 (19.3 %) crossbreds (Table 2).

Considering just the 751 incident patellar luxation cases, information was available on which limb was affected for 722 (96.1 %) dogs. Of these, 273 (27.8 %) were bilateral and 449 (62.2 %) were unilateral (245 (33.9 %) for the left stifle and 204 (28.3 %) for the right stifle). Medical management was used in 293/751 (39.0 %) of the incident cases, with the remainder receiving no medical treatment. Surgical intervention was recorded in 99/751 (13.2 %) of the incident cases, with the remainder not having surgery during the study period. The median age at the first recorded surgery for these 99 dogs was 2.9 years (IQR 1.5-5.6, range 0.4–13.1) and the median period from first diagnosis to first surgical intervention was 15 days (IQR 5–43, range 0–901). Of the incident cases, 28/751 (3.7 %) were referred for further case management.

As described above, three variables (*purebred, Kennel Club breed group* and *adult bodyweight)* were not considered in multivariable modelling so the univariable results alone are reported here. No association was shown between purebred status and patellar luxation: purebred dogs had 1.1 times the odds (95 % CI 1.0–1.3, *P* = 0.133) compared with crossbred dogs. Of the Kennel Club breed groups, Toy (OR 3.7, 95 % CI 3.1–4.3, *P* < 0.001) and Utility (OR 1.6, 95 % CI 1.2–2.0, *P* < 0.001) showed higher odds of patellar luxation compared with Kennel Club non-recognised dogs. Dogs of adult bodyweight < 10.0 kg had 2.4 times the odds (95 % CI 2.3–3.3, *P* < 0.001) compared with dogs weighing 10.0–19.9 kg (Table 2).

Univariable logistic regression modelling identified liberal associations (*P* < 0.20) with patellar luxation for six variables: (*breed, breed-relative bodyweight, age, sex, neuter* and *insurance*) (Table 2). Following evaluation using multivariable logistic regression, the final model comprised six risk factors: *breed, breed-relative bodyweight, age, sex, neuter* and *insurance*. No biologically significant interactions were identified. The final model was improved by inclusion of the clinic attended as a random effect and these results are reported (*P* < 0.001, rho = 0.0.028 indicating that the clinic attended accounted for 2.8 % of variation). The final non-clustered model showed good fit and discrimination (area under the ROC curve: 0.818, Hosmer-Lemeshow test: *P* = 0.119). After accounting for the effects of the other variables evaluated, 11 breeds showed increased odds of patellar luxation diagnosis compared with crossbred dogs. The breeds with the highest odds included the Pomeranian (OR: 6.5, 95 % CI 4.0–10.7, *P* < 0.001), Chihuahua (OR: 5.9, 95 % CI 4.4–7.9, *P* < 0.001), Yorkshire Terrier (OR: 5.5, 95 % CI 4.3–7.1, *P* < 0.001) and French Bulldog (OR: 5.4, 95 % CI 3.1–9.3, *P* < 0.001). Dogs with bodyweight below their breed mean had a 1.4 times odds of diagnosis (95 % CI 1.2–1.6, *P* < 0.001) compared with dogs weighing at or above their breed mean. Dogs aged ≥ 12.0 years showed 0.4 times the odds (95 % CI 0.3–0.5, *P* < 0.001) of an incident patellar luxation compared with dogs aged < 3.0 years. Females had 1.3 times the odds (95 % CI 1.1–1.4, *P* = 0.003) (95 % CI 1.1–1.5, *P* < 0.001) of patellar luxation compared with males. Neutered dogs had 2.4 (95 % CI 1.8–3.2, *P* < 0.001) and insured dogs had 1.9 (95 % CI 1.6–2.3, *P* < 0.001) times the odds of patellar luxation compared with entire and uninsured dogs respectively (Table 3).

**Table 3:** Final mixed-effects multivariable logistic regression model for risk factors associated with a diagnosis of patellar luxation in dogs attending primary-care veterinary practices in England

Variable	Category	Odds Ratio	95 % CI	*P*-value
Breed	Crossbred	Base		
	Other breed-types	0.4	0.3–0.5	<0.001
	Bichon	3.0	2.0–4.4	<0.001
	Bulldog	2.9	1.7–5.0	<0.001
	French Bulldog	5.4	3.1–9.3	<0.001
	Chihuahua	5.9	4.4–7.9	<0.001
	Lhasa Apso	3.1	1.9–5.1	<0.001
	Pomeranian	6.5	4.0–10.7	<0.001
	Pug	3.7	2.3–5.8	<0.001
	Shih-tzu	1.2	0.8–2.0	0.392
	Cavalier King Charles Spaniel	2.5	1.8–3.5	<0.001
	Jack Russell Terrier	1.5	1.1–2.0	0.008
	Staffordshire Bull Terrier	0.5	0.3–0.7	0.001
	West Highland White Terrier	2.0	1.4–2.8	<0.001
	Yorkshire Terrier	5.5	4.3–7.1	<0.001
Breed relative bodyweight^a^	At or above breed mean	Base		
	Below breed mean	1.4	1.2–1.6	<0.001
	No recorded weight	0.3	0.2–0.4	<0.001
Age (years)	<3.0	Base		
	3.0 - <6.0	0.8	0.7–1.0	0.019
	6.0 - <9.0	0.8	0.6–1.0	0.017
	9.0 - <12.0	0.7	0.5–0.8	0.001
	> or = 12.0	0.4	0.3–0.5	<0.001
	No age available	158.7	114.8–219.5	<0.001
Sex	Male	Base		
	Female	1.3	1.1–1.5	<0.001
	Unknown	0.1	0.0–0.9	0.038
Neutered	Entire	Base		
	Neutered	2.4	1.8–3.2	<0.001
	Unknown	1.2	0.9–1.6	0.222
Insured	Uninsured	Base		
	Insured	1.9	1.6–2.3	<0.001
	Unknown	1.1	0.9–1.3	0.488

Breed relative bodyweight^a^ for maximum bodyweight recorded in dogs aged over 18 months compared to the mean bodyweight of all study dogs for breed and sex

## Discussion

This is the largest primary-care veterinary study to provide epidemiological data on canine patellar luxation to date. Over recent years, there has been much discussion of the need for reliable prevalence data on canine disorders in order to prioritise health reforms and this paper aimed to fulfil part of this need [41–43]. A prevalence of 1.3 % was shown here for patellar luxation diagnosis overall from a population of 210,824 dogs attending 119 clinics in England. Smaller-size breeds such as the Pomeranian, Chihuahua, Yorkshire Terrier and French Bulldog were at increased odds. Almost 40 % of patellar luxation cases received some medical management while 13 % were managed surgically and 4 % were referred for further case management. These results identified that patellar luxation is a relatively common disorder in dogs in England and has non-trivial welfare impacts on affected individuals.

The 1.3 % prevalence for patellar luxation diagnosis reported in the current study is similar to a previous publication from a US referral population that identified a 1.5 % prevalence [5] but is significantly lower than the 8 % prevalence reported in the 1970s [21]. These studies differed in methodologies and denominator populations and it is now considered that primary-care veterinary data may be more representative of the general dog population for prevalence estimation than referral data [23, 24]. However, it should also be noted that the application of primary-care veterinary data for research relies heavily on the accuracy and completeness of the clinical care notes and this can be affected by differing clinical examination and diagnostic protocols and variable recording of clinical conditions across veterinary primary-care clinics.

The current study substantiated previously reported breed-related variation in the prevalence of patellar luxation [3–5, 21]. Breeds that were commonly affected in the current study included the Pomeranian (6.5 % prevalence of diagnosis), Yorkshire Terrier (5.4 %) and Chihuahua (4.9 %) have also been reported as predisposed in previous publications. The OFA prevalence statistics on patellar luxation identify the Pomeranian (37 % prevalence in submissions) and Yorkshire Terrier (24 %) as the most affected breeds submitted [30]. The higher prevalence reported by the OFA in those breeds could be indicative of differences between the predominantly North American population in OFA compared with the English dogs in VetCompass but may also reflect differences in diagnostic criteria and diagnostic sensitivity between the data sources as well as uncertainly about the directions of selection biases in the OFA data due to the voluntary nature of case submissions. Interestingly, the current study does not support recent reports in the literature that suggest that dogs of large bodysize, and the Labrador Retriever in particular, have a high prevalence of patellar luxation [7, 10, 26, 27]. These earlier studies may have suffered referral bias towards certain breeds that are regularly referred, and by high levels of Labrador Retriever ownership in general.

The high prevalence of diagnosed patellar luxation in certain breeds reported in this study (Table 2), along with increased odds of patellar luxation diagnosis demonstrated in 11 breeds after accounting for other demographic factors, supports current thinking of a breed-related and therefore heritable basis for patellar luxation [5, 27]. Selective breeding for erstwhile desirable body conformations may have non-intentionally co-selected for risk factors that also promote patellar luxation; these may include musculoskeletal deformities such as tibial torsion [44], femoral varus [45], patella positioning [46] or femoral head inclination [26]. Pomeranians with more severe forms of patellar luxation have been shown to have increasing complexity of limb deformity including increased femoral varus [45]. Genomic analyses have identified loci on certain chromosomes implicated in patellar luxation in specific dog breeds such as the Dutch Flat-coated Retriever [47] and Pomeranian [48], with chromosome seven being of particular interest. These genetic studies, along with epidemiological analyses such as presented in the current study, highlight an opportunity to affect the prevalence and severity of patellar luxation in predisposed breeds using strategic breeding programmes underpinned by sound epidemiological evidence.

Consistent with the majority of previous studies [10, 21, 27], females were over-represented for patellar luxation in the current study, showing 1.3 times the odds of males. This association with females has previously been reported to also extend to the severity of disease, with females dogs having greater patella-femoral cartilage erosion than male dogs [25] although severity was not evaluated in the current work. Neuter status was also a significant risk factor in the current study, with neutered dogs having 2.4 times the odds of patellar luxation compared with entire animals. A study of small and miniature breed dogs in Austria similarly reported that neutered dogs had 3.1 times the odds of being affected with patellar luxation [49]. Although, the pathogenesis behind these sex and neuter associations is currently unclear, hormonal influences on growth rates and musculoskeletal development are possible. Oestradiol has been shown to promote development of a shallower trochlear sulcus [3] and hypoestrogenemia associated with ovariohysterectomy has been implicated in other orthopaedic conditions of the stifle such as cranial cruciate ligament disease [50]. Interestingly, although neutering is often associated with an increase in bodyweight [51], the results from the multivariable model indicated reduced odds of patellar luxation for dogs of higher than average adult bodyweight for their breed and sex, suggesting that increased bodyweight is not the mechanism by which neutering is associated with increased patellar luxation. It is also worth noting that the neuter value reflected the status at the final record and this was not necessarily the same as the neuter status when the patellar luxation disorder was first diagnosed.

The current study identified lower adult bodyweight as a significant risk factor for patellar luxation, with univariable analysis of dogs weighing under 10 kg showing 2.8 times the odds of patella luxation compared with those weighting 10.0–19.9 kg. Previous studies have also identified lower bodyweight as a risk factor, with small dogs having 12 times the odds of large dogs [21]. However, the results of the current study showed minimal differences in odds ratios between weight categories above 10 kg, suggesting that patellar luxation odds could be linked with genetic factors associated with miniaturization [21]. Breeds that were particularly affected included the Pomeranian (6.4 % prevalence of diagnosis), Yorkshire Terrier (5.3 %) and Chihuahua (4.8 %), consistent with previous publications reporting increased susceptibility in small breed dogs [4, 10, 21, 25, 27, 28]. A report on a prospective screening programme for patellar luxation in small and miniature breed dogs in Austria similarly concluded that miniaturisation may play a role in the development of patellar luxation [49].

Dog breeds are intra-species groups developed under controlled conditions by man to have relatively uniform phenotypic traits including bodysize, coat colour, conformation and behavioural characteristics [36]. Therefore, because bodyweight is an intrinsic characteristic for each breed, this study avoided including bodyweight values into the multivariable modelling as this would have removed bodyweight effects from the breed-related results. However, in order to still explore multivariable association between bodyweight and patellar luxation, a breed-relative bodyweight variable was included in multivariable modelling to describe each dog's adult bodyweight in relation to its individual breed and mean. Multivariable analysis demonstrated that, after accounting for the other factors evaluated, dogs weighing below their breed average bodyweight had 1.4 times the odds of patellar luxation compared with dogs weighing at or above their breed mean. One possibility for this finding could be that lighter dogs have reduced muscle mass overall, including reduced quadriceps muscle mass, which promotes increased patella laxity [10, 28]. However, an inverse direction of causality is also possible, where patella luxation may cause reduced limb usage over a prolonged period and therefore lead to muscle atrophy and lower bodyweight.

A general tendency towards increased diagnostic rates in insured individuals has previously been reported in dogs for disorders including chronic kidney disease [52], mast cell tumour [53] and cranial cruciate ligament disease [54]. Differential recorded diagnostic rates between insured and uninsured animals may reflect relaxation of financial constraints and higher expectations of clinical care by owners that encourage more thorough record-keeping and more extensive clinical investigations on insured animals [55]. Although insured dogs showed almost twice the odds of patellar luxation diagnosis compared with uninsured dogs in the current study, an earlier study identified a four-fold effect for insurance in diagnosis of cranial cruciate ligament disease [54]. The differing effects of insurance between the disorders may be explained by a greater confidence with which patellar luxation may be diagnosed in the conscious dog during clinical examination alone without necessarily requiring expensive further investigation compared with cruciate ligament disease which may require stifle stability examinations under anesthetic and radiography for diagnosis [54].

The current study reported that fewer than 30 % of patellar luxation cases were bilateral. Previous reports in a referral population have reported higher levels of bilateral luxation, typically around 50 % [4, 10, 27]. Increased bilateral luxation diagnosis in the referral caseloads may reflect differing completeness of diagnosis and clinical recording between primary-care and orthopaedic second opinion consultations because of differing clinician experience or time available for evaluation, but could also reflect effects of referral bias where more severely affected cases (i.e., bilaterally affected animals) are more likely to be referred.

In terms of clinical management, 39 % of dogs diagnosed with patellar luxation in the current study received some medical treatment while the remainder did not receive any direct therapy for the condition during the study period. Although patellar luxation may initially cause minimal discomfort [49], medical treatment can be required later to manage secondary osteoarthritis [6] and the disorder has also been associated with increased risk of cranial cruciate ligament disease [56]. Surgical stabilisation was performed in just 13 % of the primary-care patellar luxation cases in the current study compared with 83 % of referred patellar luxation cases that are reported to receive surgical intervention [10]. It is generally recommended to avoid surgery on clinically asymptomatic patellar luxation which may have been diagnosed on palpation-manipulation alone without any history of lameness [4]. The lower surgical rate reported in the current primary-care study may be a better reflection of the true impact of the disease than can be gained from the referral caseloads, but it is also possible that primary-care clinical management is not fully availing of the benefits from surgery to provide improved clinical outcomes [3, 6, 57]. Despite the relatively low primary-care surgical uptake, the median period from diagnosis to surgery in the current study was short, at around 2 weeks, for those dogs that did receive surgery. This suggests that when surgery was chosen, it was generally a clear-cut decision. Primary-care surgically managed dogs also tended to be young, averaging around 3 years of age. The high level of primary-care dogs diagnosed with patellar luxation that received no medical or surgical management may reflect a preponderance of incidental, non-clinically affected patellar luxation identified in primary-care practice whereas referral caseloads by definition have been pre-selected as more severely affected and therefore potential surgical candidates, further cementing primary-care clinical data as the more representative perspective on the true spectrum of clinical health in companion animals overall.

Referral rates for patellar luxation in the current study were relatively low, with just 4 % of patellar luxation cases referred compared with the 21 % referral rate reported previously for cranial cruciate ligament disease [54]. The low referral rate for patellar luxation may reflect perceptions by veterinarians that complete diagnosis and management is achievable in the primary-care setting. Surgical techniques for patellar luxation may be considered relatively straightforward, even with the expertise and more-limited inventory of surgical equipment [4] often available in primary-care practices. However, complication rates as high as 18-29 % have been reported following patellar luxation surgery, suggesting that this type of surgery should be regarded as neither simple nor low risk [7, 14].

From a welfare perspective, diseases which are present from a young age and do not substantially shorten lifespan can represent a significant welfare burden for affected animals due to their chronicity, especially if their prevalence and severity is high [58]. Patellar luxation is a developmental disease, not being present at birth but developing from a young age [4, 5] and hence clinical signs may often only become evident as the animal grows [21]. This study reported that a median age at first diagnosis of patellar luxation of 4 years and that dogs aged over 9 years had significantly lower odds of first diagnosis compared with dogs aged under 3 years. These findings that patellar luxation commonly affects dogs from an early age are in agreement with other studies [10, 26–28]. Although patellar luxation can be asymptomatic during early life [3, 4], subsequent development of osteoarthritis, pain and lameness is possible [6–8, 28, 49].

Although this study addressed many of the selection and recall biases that have limited previous referral and questionnaire study methods, some limitations still remained. The practices that contributed to this study were all private, primary-care practices mainly based in central and south-eastern England and it is possible that patient populations at charity clinics or practices based elsewhere in the country might apply differing diagnostic processes and clinical management. Any such differences may affect generalisability of the descriptive prevalence results which are more heavily dependent on prevailing levels of clinical care but should have minimal impact on the risk factor results which are more dependent on basic physiology and therefore will be more constant across all dogs in England [59]. The study relied on the primary-care veterinarian to accurately diagnose and record the presence of patellar luxation and it is possible that some affected dogs were misclassified or that their diagnosis was not recorded. In consequence, it is important to emphasise that studies based secondary data such as primary-care veterinary clinical data or insurance records that were not primarily collected for research purposes will report the apparent prevalence of diagnosed disorders rather than the true prevalence of all existing disorders [31]. It is also possible that manipulation of the patellae may be more frequently performed as part of the general physical examination in toy breeds in comparison to other breeds, which may bias toy breeds towards showing relatively higher proportional patellar luxation compared with larger breeds. Data were not available in the current study to describe the direction of luxation (medial, lateral, both) or the grading of patellar luxation (i.e., grades I-IV/IV) in individual cases. Although this did not affect the breed prevalence and risk factors for patellar luxation overall because all patellar luxation cases were included regardless of directionality, it would have been interesting to explore the relative preponderance of and risk factors for the direction and severity of luxation cases.

## Conclusions

The results of this study highlight that 1.3 % of dogs overall in England are affected by patellar luxation and that there are strong breed predispositions towards certain small breeds of dog, particularly the Pomeranian and Yorkshire Terrier. Being neutered, female and weighing less than the breed mean were additional risk factors for patellar luxation. Forty per cent of cases received active medical treatment and 13 % received surgical intervention, suggesting that patellar luxation should be included a welfare priority for dogs and that control strategies for this disorder should be considered as worthwhile breeding goals, especially in predisposed breeds.

## Abbreviations

CI, confidence interval; EPR, electronic patient record; IQR, interquartile range; OFA, Orthopaedic Foundation of America; OR, odds ratio; VMDB, Veterinary Medical Database.
